# Endoscopic-Ultrasound-Guided Fine-Needle Aspiration and the Role of the Cytopathologist in Solid Pancreatic Lesion Diagnosis

**DOI:** 10.1155/2012/317167

**Published:** 2012-05-15

**Authors:** Shahzad Iqbal, David Friedel, Mala Gupta, Lorna Ogden, Stavros N. Stavropoulos

**Affiliations:** ^1^Division of Gastroenterology, Department of Medicine, Winthrop-University Hospital, Mineola, NY 1150, USA; ^2^Department of Pathology, Winthrop-University Hospital, Mineola, NY 1150, USA

## Abstract

Endoscopic ultrasound (EUS) is the most sensitive imaging modality for solid pancreatic lesions. The specificity, however, is low (about 75%). It can be increased to 100% with an accuracy of 95% by the addition of fine-needle aspiration (FNA). Cytopathology plays an important role. The final diagnosis is based upon the correlation of clinical, EUS, and cytologic features. A close interaction with the cytopathologist is required in improving the diagnostic yield. In this paper, we present an overview of the role of EUS-guided FNA and importance of close interaction with the cytopathologist. Day to day examples of different solid pancreatic lesions have been presented at the end.

## 1. Introduction

Endoscopic ultrasound (EUS) is an emerging imaging modality. EUS-guided fine-needle aspiration (FNA) plays an important role in solid pancreatic lesions. A close interaction with cytopathology is vital in improving the diagnostic yield. The final diagnosis is based upon correlation of clinical, EUS, and cytologic features. In this paper, we will discuss the role of EUS-FNA, and the importance of cytopathology in the diagnosis of solid pancreatic lesions. We will describe the history and safety of EUS, indications for an EUS-FNA, and a short description of the technique of EUS-FNA. We will also discuss the importance of arranging an onsite cytopathologist and alternatives if that is not feasible. Finally, we will present the clinical, EUS, and key cytologic features of a few representative solid pancreatic lesions. 

## 2. Endoscopic Ultrasound (EUS): Background

**Figure 1:**
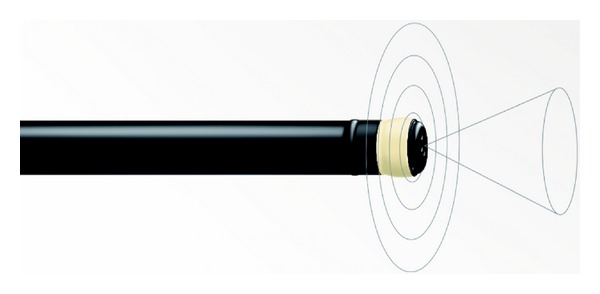
Radial echoendoscope. The tip of the scope scans perpendicular to its axis, providing 360-degree view.

**Figure 2:**
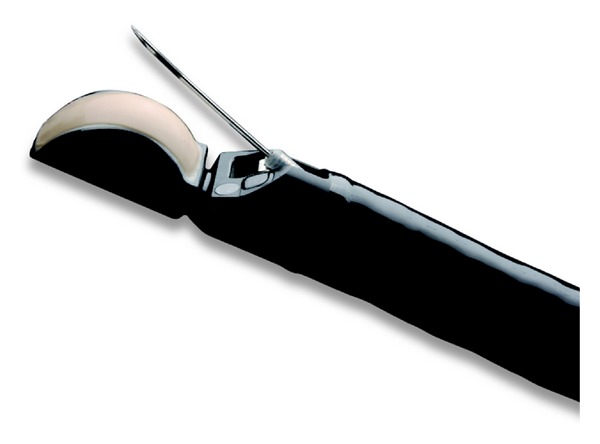
Linear echoendoscope. The tip scans parallel to its longitudinal axis. An FNA needle is seen coming out of the scope channel.

Endoscopic ultrasound (EUS) was first introduced by Dr. Eugene DiMagno in the 1980s by combining a high-frequency ultrasound transducer to an endoscope [1]. Initial echoendoscopes were radial, which scan perpendicular to scope's axis and provide 360-degree images similar to computerized tomography (CT) (Figure 1). In 1991, convex linear-array echoendoscope was introduced by Pentax (FG-32). These linear scopes scan parallel to the longitudinal axis of the scope and enable fine needle aspiration (FNA) and different therapeutic applications (Figure 2). 

Different imaging modalities are available to help diagnose solid pancreatic lesions including transabdominal ultrasound, computerized tomography (CT), magnetic resonance imaging (MRI), endoscopic retrograde cholangiopancreatography (ERCP), EUS, and positron emission tomography (PET). EUS is considered one of the most sensitive imaging modalities to detect pancreatic masses, with an accuracy of 78–94% for T (local tumor) stage and 64–82% for N (lymph node) stage [2–4]. EUS is an outpatient procedure and can be done under conscious sedation, like a standard upper endoscopy. The pancreas is imaged from the stomach and duodenum.

Overall, EUS is a safe procedure with most of the complications related only to FNA. The complications include bleeding (0–1.3%) [5–7], perforation 0–0.4% [5, 6], infection (0.3%) [5, 6], and pancreatitis (1-2%) [5]. The risk of bacteremia is low, and prophylactic antibiotics are not recommended except for EUS-FNA of pancreatic cystic lesions [8]. The risk of tumor seeding is significantly lower as compared to percutaneous approach [9] with only four case reports so far. The risk of tumor seeding is further diminished due to the inclusion of needle tract in the resection field of pancreatic head lesions.

## 3. EUS-Guided Fine Needle Aspiration (EUS-FNA): Indications, Accuracy, and Technique

Although EUS is a very sensitive imaging modality, its ability to differentiate benign inflammatory and malignant pancreatic masses is low. The specificity is only about 75% [10]. The specificity can be increased to 100% with FNA with an accuracy of 95% [11]. However, the negative predictive value of EUS-FNA is low (56%) [12], and a negative result does not completely exclude malignancy. Hence, the need for routine EUS-FNA of potentially resectable pancreatic mass lesions noted on other imaging modalities is controversial [13]. In a review article by Varadarajulu and Eloubeidi [14], EUS-FNA was indicated in the following cases.

1. Unresectable mass as a prerequisite for adjuvant chemotherapy or radiation.
2. Suspected other tumor types like lymphoma, small cell metastasis, or neuroendocrine tumors that require different therapy.
3. When the pretest probability of malignancy is low.
4. Patient refuses major surgery without a definitive diagnosis.

**Figure 3:**
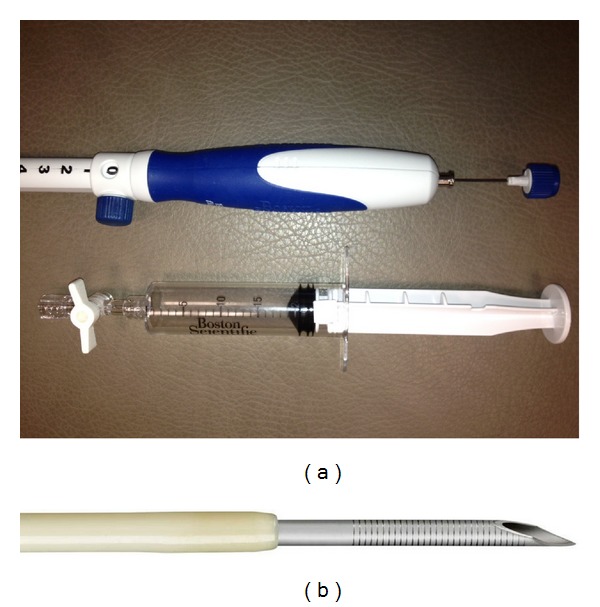
EUS-FNA needle. (a) The handle of the FNA needle is shown with a stylet inside and a suction syringe. (b) The tip of the FNA needle used for puncturing the tissue is shown.

**Figure 4:**
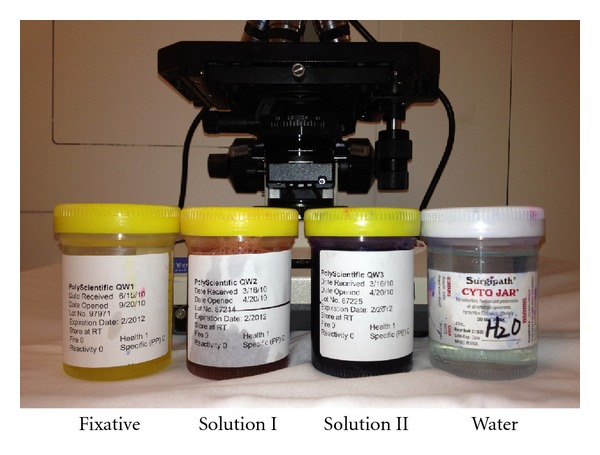
Diff-Quik staining method. Air-dried smears are stained in three steps and takes about 10–20 seconds each. The smear is first fixed in methyl alcohol solution, followed by staining with solution I containing L-xanthene (an eosin variant), and then solution II containing methylene blue and azure A. The slide is then rinsed with water and the wet slide is viewed under the microscope.

EUS-FNA is done under real-time EUS imaging [15]. The needle device is inserted into the biopsy channel of the linear echoendoscope. The stylet is withdrawn a few millimeters to expose the sharp tip of the needle, followed by advancement of the tip into the target lesion (Figure 3). Doppler is used to avoid any vessels. Once the needle is inside the target tissue, the stylet is first pushed in to expel any needle tract tissue and then removed out of the needle. Suction is applied by attaching a 10–20 mL syringe. The needle is moved to and fro about 10–20 times in a fanlike pattern inside the lesion. The suction syringe is released, the needle is withdrawn into the catheter, and the whole device is removed from the echoendoscope. The material is sprayed onto glass slides by using either air-filled syringe or the stylet, with half of the material fixed in ethanol (for Papanicolaou staining) and the remainder air-dried for onsite cytologic evaluation using Diff-Quik staining (Figure 4). Material for cell block is then obtained by recovering tissue fragments or clot from slides and also rinsing the EUS needle with sterile saline. Pancreatic adenocarcinoma and lymph node metastases of known origin can be usually diagnosed with smears alone. However with suspected neuroendocrine tumor, metastasis of unknown primary, mesenchymal neoplasms, lymphoproliferative disorders, or any other unusual case, sufficient material is needed in cell block for immunocytochemistry [16]. With suspected lymphoma, a separate sample in Roswell Park Memorial Institute (RPMI) solution is preferred for flow cytometry [17].

## 4. Diagnostic Yield of EUS-FNA and the Importance of an Onsite Cytopathologist

The diagnostic yield of EUS-FNA for solid pancreatic lesion is 86.8–98.5% for cytologic and 68.9–89% for histologic examinations, while 65–100% for lymph nodes [18]. However, there are certain variables that can affect the accuracy and diagnostic yield of EUS-FNA. These can be categorized as either endoscopist or cytopathologist related.

The success rate of EUS-FNA increases with endoscopist experience. The American Society of Gastrointestinal Endoscopy (ASGE) has recommended at least 150 supervised EUS procedures, including 50 EUS-FNAs (of which half are pancreatic and another half nonpancreatic) to achieve comprehensive competency [19]. Getting the target lesion in good view, selecting proper needle size, and proper FNA technique are associated with increased diagnostic yield [20]. The available needles are 25, 22, and 19 gauges. For solid pancreatic lesions, the 25-gauge needle is at least equivalent [21–24] or even superior for cytological diagnosis [25, 26] due to less blood contamination as compared to the standard 22-gauge needle. However, certain tumors such as malignant lymphomas, rare pancreatic neoplasms, well-differentiated adenocarcinoma, mesenchymal tumors, as well as benign conditions, such as autoimmune pancreatitis may be difficult to diagnose based on cytology alone. In such cases, core biopsies can be obtained using 22- or 19-gauge aspiration needles [27]. A spring-loaded 19-gauge Tru-Cut needle is also available for histologic diagnosis. However, it is difficult to deploy especially for pancreatic head lesions [28].

The diagnostic success of EUS-FNA also depends on the cytopathologist's experience. Unlike percutaneous biopsies, EUS-FNA material is contaminated by gastrointestinal epithelium [29, 30] leading to errors in diagnosis. It is important for the cytopathologist to be aware of the route traversed by the needle for proper evaluation of the smears. Training courses are required for cytopathologist with no previous experience with EUS-FNA [31, 32]. Proper handling and triaging of EUS-FNA material following immediate onsite cytologic evaluation are very helpful [33]. Immediate cytologic evaluation not only improves the diagnostic yield, but can potentially reduce the number of needle passes, procedure time, and patient risk [34]. However, it is expensive and many of the centers may not have an onsite cytopathologist. In a study by Savoy et al. [35], even experienced endosonographers were less reliable than cytotechnologists in evaluating onsite adequacy macroscopically. When either an onsite cytopathologist or cytotechnologist is not available, options are to use multiple needle passes or telecytopathology.

The optimal number of EUS FNA needle passes range from two to six [36]. In study by Erickson et al. [34] where onsite cytopathologist was not available, 5-6 passes were needed for diagnosis of pancreatic masses and 2-3 for liver metastases and lymph nodes. However in another study [36], at least 7 passes were needed for pancreatic and miscellaneous masses (including mediastinal, perirectal, and subepithelial) and 5 passes into lymph nodes for correct diagnosis. In a retrospective multicenter study [37], gross or macroscopic assessment of the specimen for adequacy by the endosonographer (“one or more small-core biopsy cylinders”) resulted in limitation of the number of needle passes to only 1-2 in 92% of the patients with solid pancreatic lesions. However, the macroscopic assessment was wrong in 13.5% of the cases due to presence of fibrous tissue, gastrointestinal contamination, or occasionally clot. However in another study [38], neither endosonographers nor cytotechnologists were able to provide a reliable assessment of FNA adequacy for solid pancreatic lesions by using gross visual inspection of the specimen on a slide. Another emerging concept is telecytopathology which may be a valid substitute to onsite cytopathologic evaluation. The slides are initially prepared and prescreened by a cytotechnologist or pathology resident and then analyzed by an offsite cytopathologist using real-time remotely operated system (MedMicro System; Trestle Corporation of Irvine, CA). In a retrospective study [39], telecytopathology was statistically equivalent in accuracy to onsite cytopathologist for pancreatic carcinoma. 

## 5. EUS and Cytologic Features of Different Pancreatic Solid Lesions

The clinical and radiological findings should correlate with the FNA results on whether the pancreatic lesion is benign or malignant [40]. It is the composite picture of cell type, microarchitecture, and nuclear and cytoplasmic features that determine the cytologic diagnosis. The smear is first examined under low magnification for cellularity, cell types, and cohesiveness, followed by high-magnification examination of nuclear and cytoplasmic features. Specific nuclear features determine malignancy, whereas cytoplasmic features and microarchitecture determine differentiation. Malignant cells exhibit disorganization and discohesion (loosely aggregated or single cells). Nuclear features of malignant cells include enlarged nuclei (higher nuclear to cytoplasmic ratio), variation in nuclear size and shape from cell to cell (anisonucleosis), hyperchromasia (increase amount of chromatin), multiple or irregular nucleoli, and abnormal mitotic figures. Cytoplasmic features (mucin in adenocarcinoma and fine red granules in neuroendocrine tumors) and microarchitecture (glands or papillae) help classify the tumor. In poorly differentiated or metastatic lesions, immunohistochemical staining of the cell blocks may be needed to determine the origin.

## 6. Examples of Different Solid Pancreatic Lesions

Next, we will discuss the clinical, EUS, and cytologic features of different solid pancreatic lesions.

**Figure 5:**
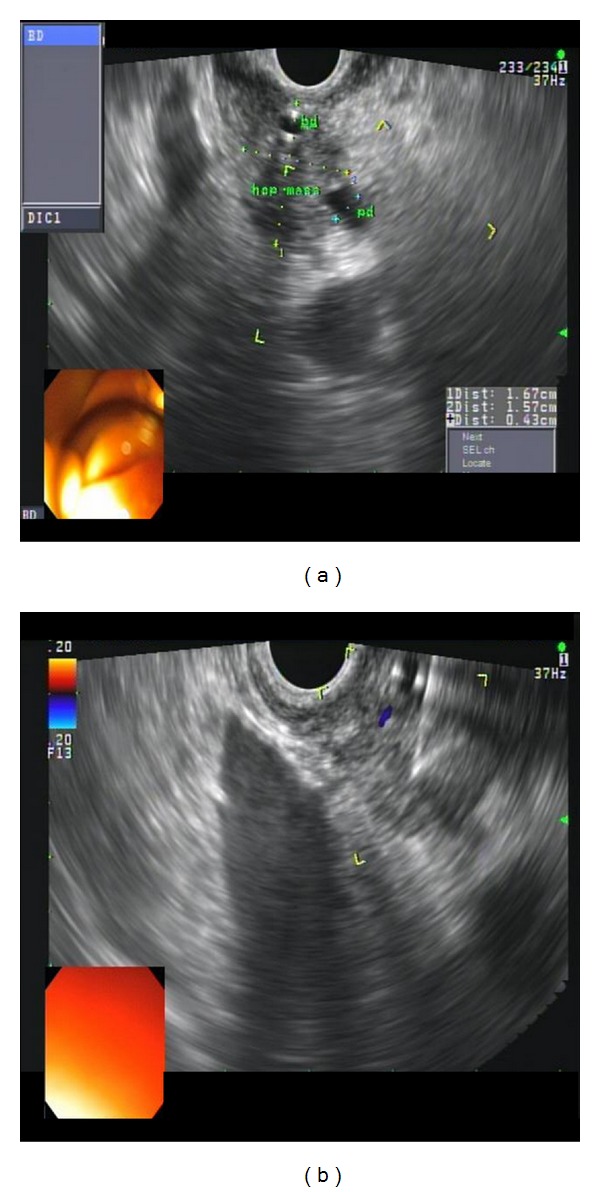
Pancreatic ductal adenocarcinoma. (a) EUS showed a 17 mm by 15 mm hypoechoic and homogenous solid mass in the pancreatic head area obstructing the distal common bile duct (CBD) and main pancreatic duct (PD) near ampulla. (b) A single FNA was done using 25-gauge needle via transduodenal approach.

(1) Pancreatic ductal adenocarcinoma: a 67-year-old gentleman presented with painless jaundice. MRI abdomen showed dilated bile and pancreatic ducts, but no obvious pancreatic mass was noted. EUS showed a 17 mm by 15 mm hypoechoic and homogenous mass in the pancreatic head area obstructing the distal common bile duct (CBD) and main pancreatic duct (PD) near ampulla (Figure 5). One FNA was done using 25-gauge needle via transduodenal approach. The specimen was analyzed by an onsite cytopathologist for adequate cellularity.

**Figure 6:**
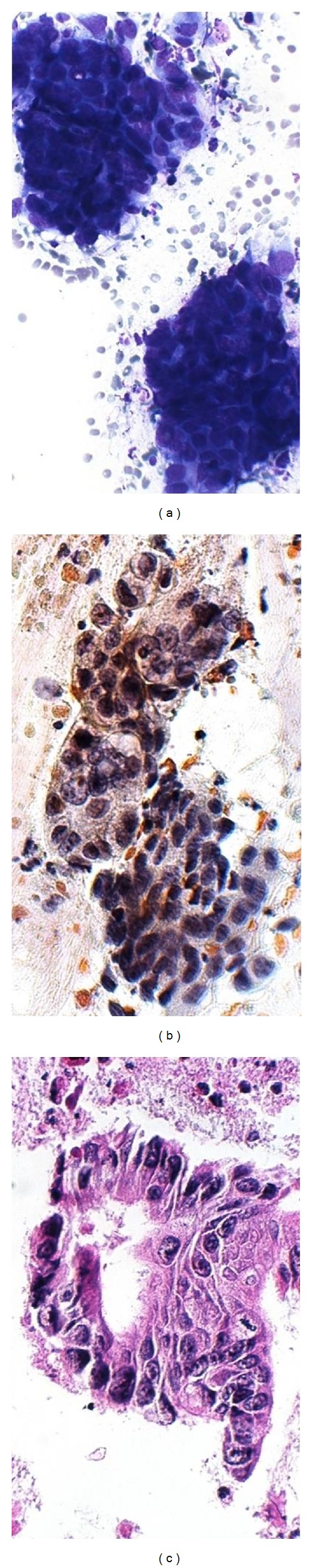
Pancreatic ductal adenocarcinoma, poorly differentiated: three-dimensional clusters in (a) Diff-Quik stain at 200x; (b) Papanicolaou's stain at 200x; (c) mitosis and hyperchromatic nuclei in cell block, H&E at 200x.

**Figure 7:**
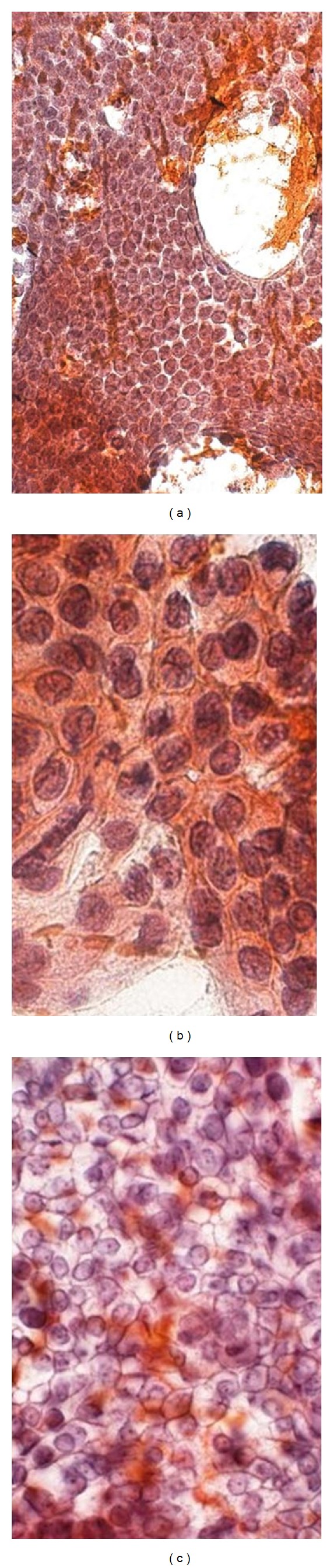
Pancreatic ductal adenocarcinoma, well-differentiated: (a) normal pancreatic ductal epithelium, Papanicolaou stain at 400x; (b) nuclear membrane irregularities, Papanicolaou stain at 600x; (c) well-differentiated adenocarcinoma with “drunken honeycombs,” Papanicolaou stain at 400x.

Onsite evaluation for a poorly differentiated adenocarcinoma is relatively easy because of increased cellularity and markedly atypical clusters with a 3-dimensional appearance (Figure 6). Necrosis may be evident in the background. On the other hand, a well-differentiated adenocarcinoma can be challenging even on alcohol fixed and Papanicolaou-stained smears. The key is to establish at least one completely normal ductal epithelium which would have uniformly sized, bland nuclei, equidistant from each other, giving it a honeycombed appearance (Figure 7). All other cell groups are compared to this normal standard. In a well-differentiated adenocarcinoma, the four most outstanding differences from normal epithelium are overall larger nuclei as compared to normal; nuclear size variation such that the largest nucleus in the group may be three times the smallest nucleus; unequal spacing of nuclei resulting in a loss of polarity, also referred to as “drunken honeycombs”; nuclear grooves and nuclear membrane irregularities [41]. If these features are not specifically sought, a false negative diagnosis may be rendered.

**Figure 8:**
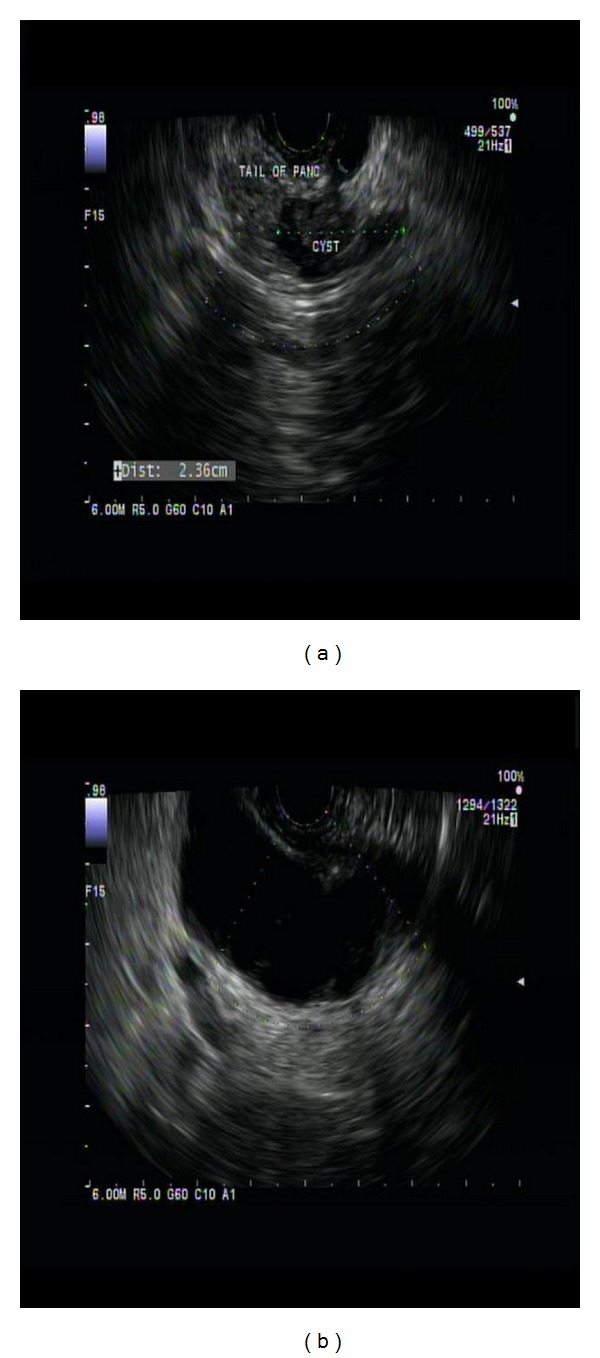
Intraductal papillary mucinous neoplasm (IPMN). (a) An irregular anechoic cystic lesion with hypoechoic solid component was noted within the tail of pancreas (suspicious for cystic neoplasm). It was communicating with the main PD. (b) The PD was itself dilated upstream of this lesion and was leading into a large anechoic pseudocyst.

(2) Intraductal papillary mucinous neoplasm (IPMN): a 68-year-old female with history of acute pancreatitis of unclear etiology and associated large pancreatic tail pseudocyst was referred for EUS evaluation and possible endoscopic drainage of the pseudocyst. EUS showed a 13 mm by 10 mm irregular anechoic cystic lesion with hypoechoic solid component within the tail of pancreas (suspicious for cystic neoplasm). It was communicating with the main PD. The PD was itself dilated upstream of this lesion and was leading into a large anechoic pseudocyst (Figure 8). One FNA pass was done of the cystic-solid lesion using 22-gauge needle via transgastric approach. The specimen was analyzed by onsite cytopathologist for adequate cellularity.

**Figure 9:**
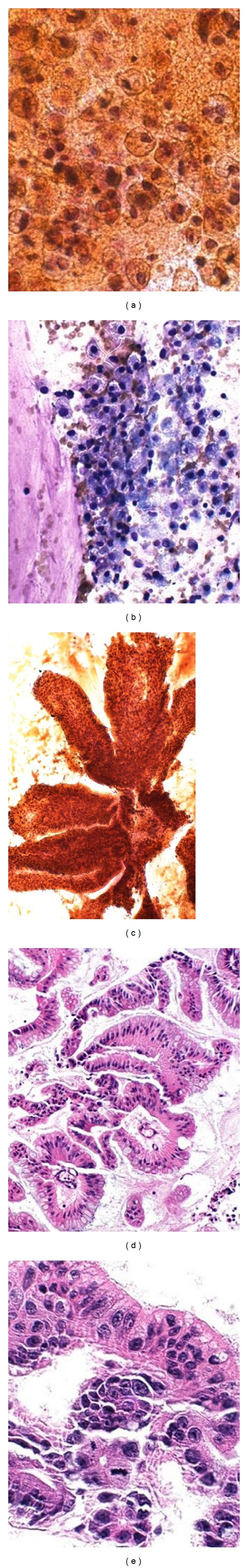
Intraductal papillary mucinous neoplasm (IPMN): Muciphages in a necrotic and mucinous background in (a) Papanicolaou stain at 400x; (b) Diff-Quik stain at 200x; Papillary fronds in (c) Papanicolaou stain at 100x; (d) cell block H&E at 200x; (e) mitosis and cytologic atypia, cell block H&E at 400x.

This case was a classic example with thick viscid mucin that did not spread easily nor air-dry readily. Microscopy revealed classic papillary fronds and numerous macrophages in a mucinous background (Figure 9). The diagnosis of IPMN is made based on the characteristic EUS findings and cytological features of viscid mucin, macrophages, and epithelium arranged in finger like papillae [42]. However, in addition, the diagnosis of in situ or invasive malignancy within the IPMN has to be evaluated separately, applying the usual criteria for malignancy. A note of caution should be added to the report that, even if no features of malignancy are present in the current aspirate smears, the lesion may still harbor malignancy in unsampled areas and, therefore, should be treated surgically.

**Figure 10:**
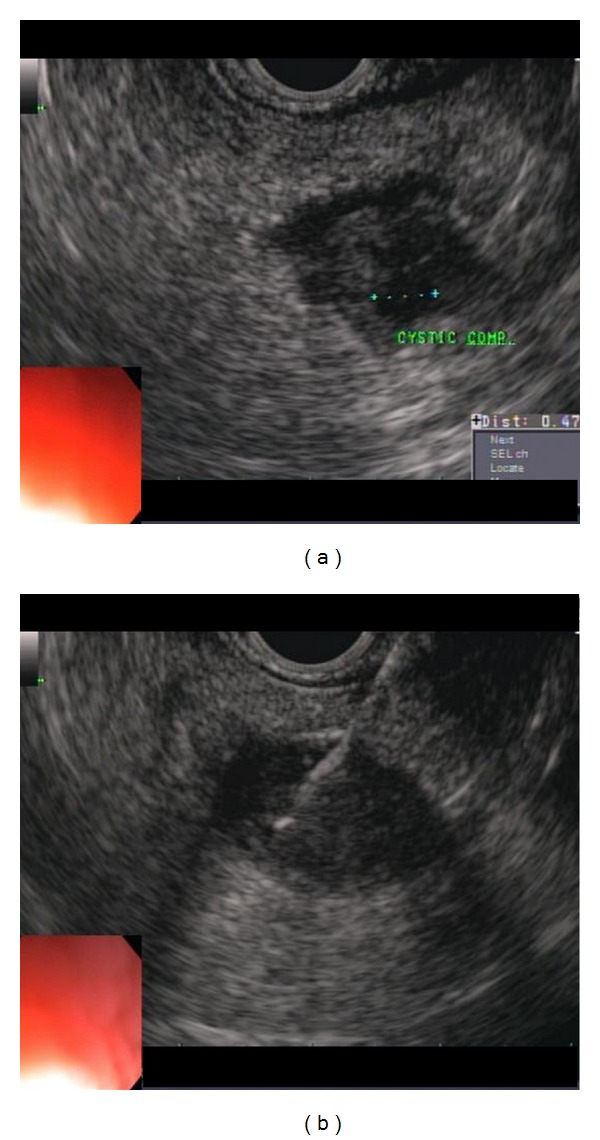
Pancreatic neuroendocrine tumor. (a) EUS showed an 18.9 mm by 9.6 mm hypoechoic solid mass with anechoic microcystic (most likely necrotic) components in body/tail of the pancreas. (b) An FNA needle is passed into the solid component via transgastric approach.

 (3) Pancreatic neuroendocrine tumor: a 48-year-old gentleman was admitted with recent onset headaches and hypertensive urgency. MRI abdomen with and without contrast showed a hypervascular 19 mm by 9 mm pancreatic body/tail solid lesion. EUS showed an 18.9 mm by 9.6 mm hypoechoic mass with anechoic microcystic (most likely necrotic) components in body/tail of the pancreas (Figure 10). Three FNA passes were obtained via transgastric approach. The specimen was analyzed by onsite cytopathologist for adequate cellularity. 

**Figure 11:**
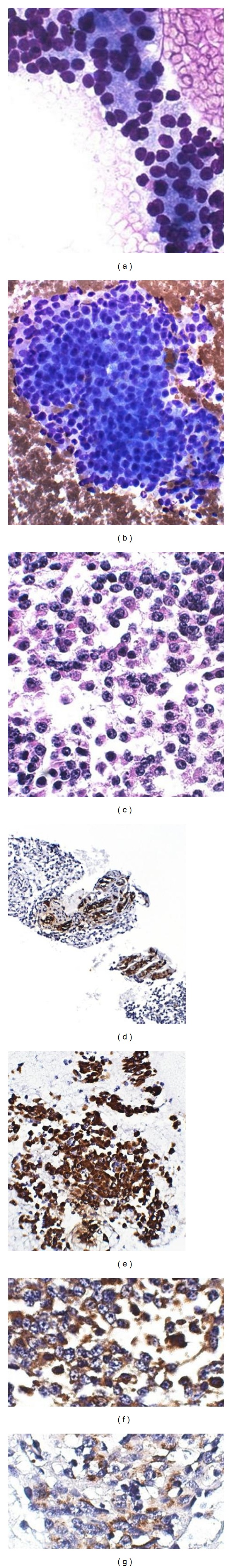
Pancreatic neuroendocrine tumor: (a) normal pancreatic acini, Diff-Quik stain at 400x; (b) cohesive 3-dimensional cluster in Diff-Quik stain at 200x; (c) discohesive cells with salt and pepper chromatin and rare pinpoint nucleolus in cell block H&E at 400x; Immunohistochemical profile of the tumor: (d) cytokeratin AE1/AE3 highlights residual ductal epithelium but not the tumor, at 100x; (e) synaptophysin, strong and diffuse positivity, at 400x; (f) positive cytokeratin Cam 5.2 at 200x; (g) positive chromogranin at 400x.

Pancreatic neuroendocrine tumors (NETs) have a variety of appearances. They can mimic normal pancreatic acinar parenchyma (Figure 11(a)), a gland forming adenocarcinoma, an acinar cell carcinoma, or even a plasmacytoma [43]. Only a small percentage show the classic “salt and pepper” nuclear detail on alcohol fixed smears and H&E of cell block. In fact, a single prominent nucleolus is not an uncommon finding. On air-dried Romanowsky-stained smears, the nuclear details are not as helpful as in alcohol fixed preparations. The key to a successful diagnosis is to keep this possibility in the differential diagnosis and obtain adequate material in cell block to do the relevant immunoperoxidase stains for neuroendocrine markers, such as chromogranin and synaptophysin (Figures 11(e) and 11(g)). Synaptophysin is more sensitive than chromogranin, but less specific. A quirky detail to be aware of is that gastrointestinal neuroendocrine tumors, including pancreatic NET, may occasionally be cytokeratin AE1 : AE3 negative. It is therefore important to include Cam 5.2, which is more reliably positive. As of now, it is not a requirement to do a Ki67 on the cell block for World Health Organization (WHO) grading based on proliferative index, but a mitotic count should be attempted if there is adequate material in the cell block [44]. As per the WHO classification of neuroendocrine tumors <2 mitosis/10 hpf is classified as a neuroendocrine tumor, 2–20 mitosis/10 hpf is classified as a well-differentiated neuroendocrine carcinoma, and >20 mitosis/10 hpf is a high-grade neuroendocrine carcinoma [45]. A note of caution: sometimes the aspiration needle may pick up incidental pancreatic endocrine microadenoma (<5 mm) or islet cell proliferations in pancreatic atrophy. Under these circumstances it is best to correlate with EUS for their relevance. 

**Figure 12:**
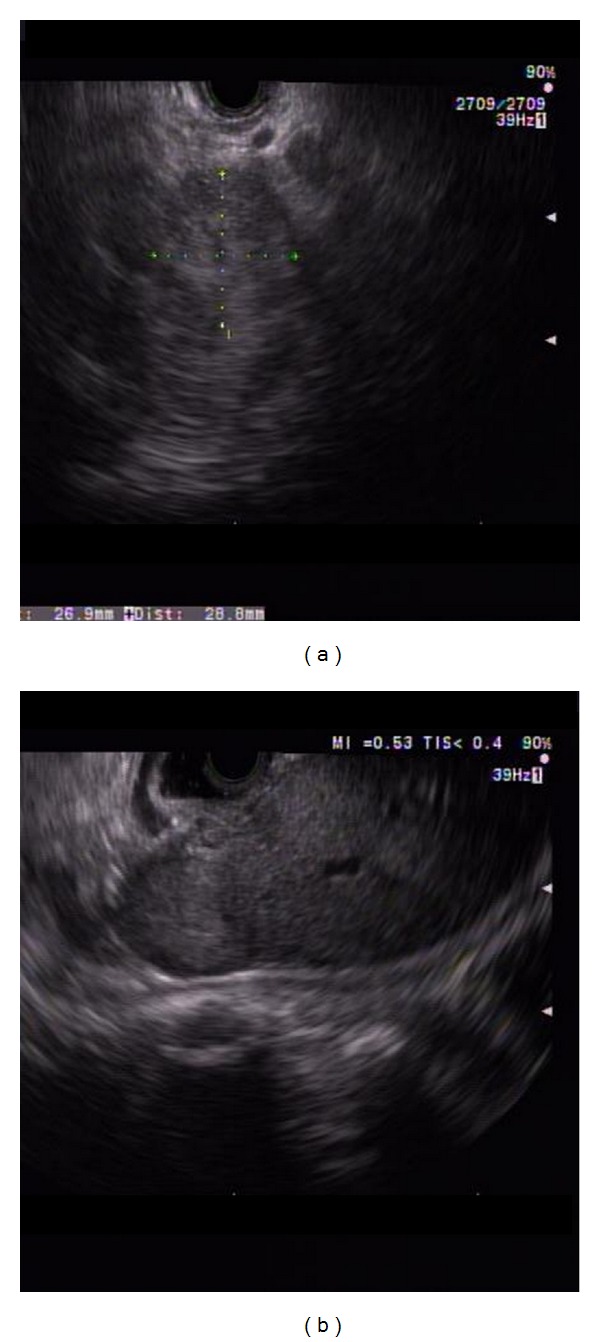
High-grade neuroendocrine carcinoma. (a) EUS showed a 29 mm by 27 mm pancreatic body hypoechoic and homogenous solid lesion. (b) Multiple solid liver lesions were also noted, consistent with metastases.

(4) High-grade neuroendocrine carcinoma: a 87-year-old gentleman status after Billroth II surgery and history of choledocholithiasis with multiple ERCP procedures in the past presented with a new onset of biliary obstruction. CT abdomen showed multiple liver lesions and retroperitoneal/portal lymphadenopathy suspicious for malignancy. EUS showed a 29 mm by 27 mm pancreatic body hypoechoic and homogenous solid lesion (Figure 12). Two FNAs passed were done via transgastric approach. The specimen was analyzed by onsite cytopathologist for adequate cellularity. Celiac lymphadenopathy and multiple liver lesions were also noted, consistent with metastases.

**Figure 13:**
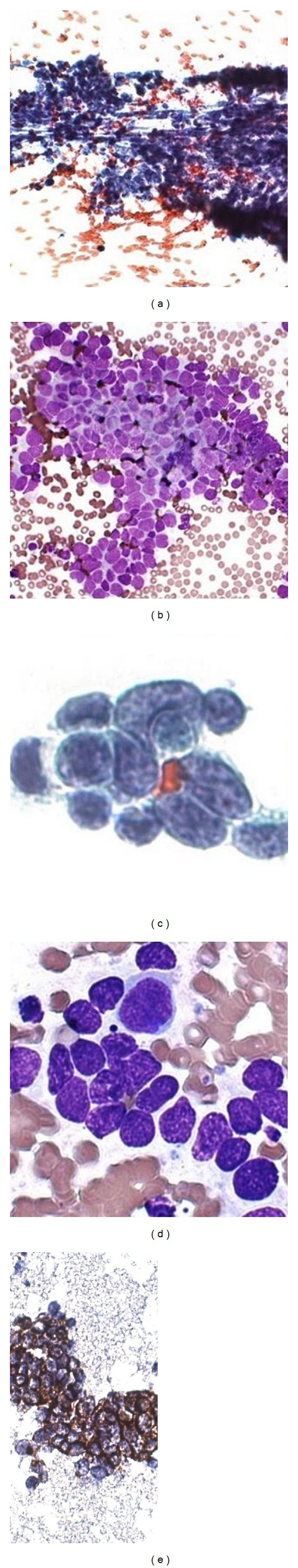
High-grade neuroendocrine carcinoma: (a) cellular smears with nuclear streaking artifact, Papanicolaou stain at 200x; (b) closely packed nuclei due to scant cytoplasm, Diff-Quik stain at 200x; (c) and (d) nuclear molding, Papanicolaou and Diff-Quik stains at 400x; (e) immunoperoxidase stain for cytokeratin Cam 5.2, at 200x.

Immediate onsite evaluation with Diff-Quik revealed abundant cellularity and necrosis with closely packed tumor cells that could be described generically as a “small, blue, round cell tumor” (Figure 13). The combination of nuclear molding, nuclear streaking artifact, mitosis, single-cell necrosis, polar cytokeratin staining, and positive neuroendocrine markers is diagnostic of a high-grade neuroendocrine carcinoma. The differential diagnosis during an onsite evaluation of these lesions includes a high-grade lymphoma. Immunoperoxidase stains, preferably on a cell block, help to sort out these two entities by a binary algorithm. Lymphoma is LCA+/CK− while high-grade neuroendocrine carcinoma is LCA−/CK+. Since high-grade neuroendocrine carcinoma occurs rarely in the pancreas, a metastasis from a lung primary should be ruled out. Clinical and chest CT findings are more important in establishing a metastasis than immunohistochemistry, because TTF1 does not reliably differentiate the two primaries as TTF1 may be positive in extrapulmonary small cell carcinoma [46]. As mentioned earlier, per WHO guidelines, a high-grade neuroendocrine carcinoma of pancreas would require a mitotic count >20/10 high power field (hpf) or a Ki-67 index >20%.

**Figure 14:**
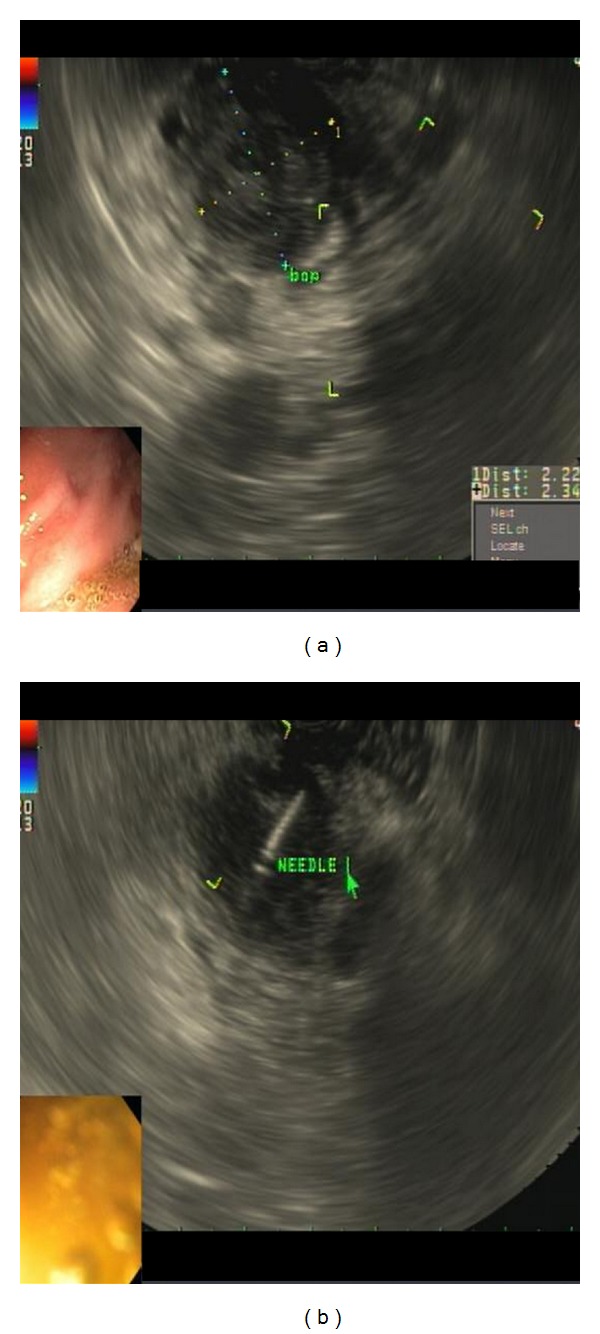
Acinar cell carcinoma: EUS showed a 25 mm by 23 mm hypoechoic solid lesion with few anechoic cystic areas (most likely necrosis) in distal pancreatic body. (b) An FNA pass is being done using 22-gauge needle via transgastric approach.

 (5) Acinar cell carcinoma: a 47-year-old female with prior history of cholecystectomy presented with epigastric pain. CT abdomen showed a 21 mm distal pancreatic body cystic lesion. EUS showed a 25 mm by 23 mm hypoechoic solid lesion with few anechoic cystic areas (most likely necrosis) in distal pancreatic body (Figure 14). Five passes were done using 22- and 25-gauge needles via transgastric approach. The specimen was analyzed by onsite cytopathologist for adequate cellularity.

**Figure 15:**
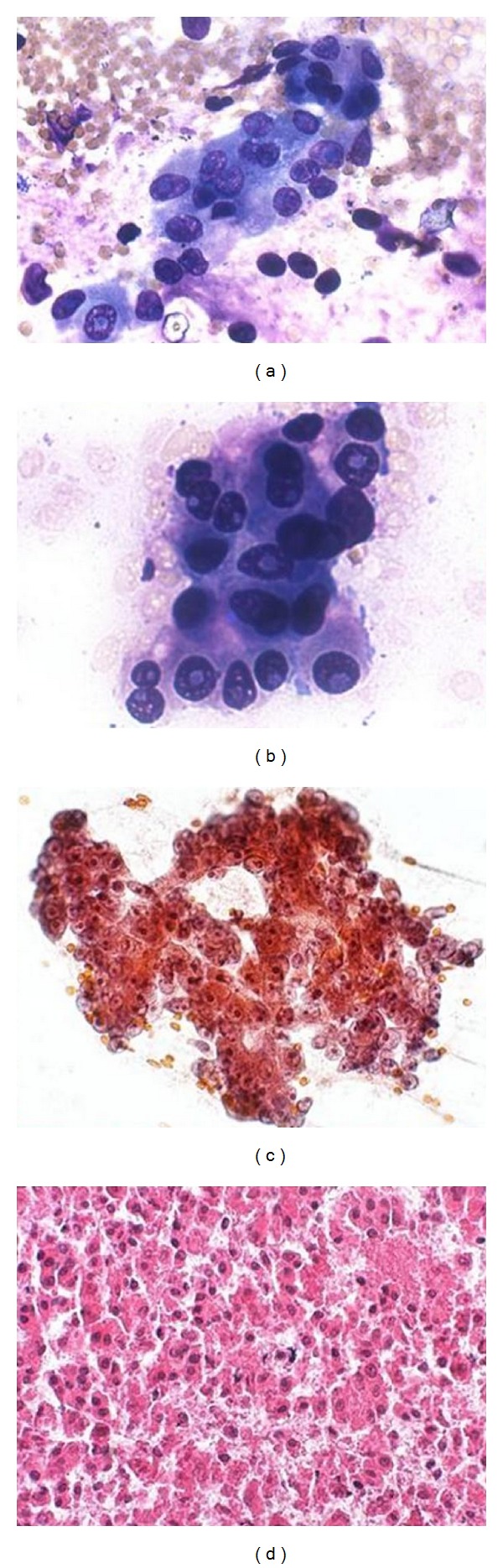
Acinar cell carcinoma: (a) one cohesive group and scattered bare nuclei, Diff-Quik stain at 400x; (b) abundant cytoplasm and prominent nucleoli, Diff-Quik stain at 600x, (c) prominent nucleoli, Papanicolaou stain at 400x; (d) discohesive cells in cell block H&E at 400x.

These tumors tend to be extremely cellular and dyscohesive on aspirate smears (Figure 15). Typically they have abundant cytoplasm and a prominent nucleolus, evident even on Diff-Quik-stained aspirate smears. Arriving at the correct final diagnosis is only possible after establishing the presence of zymogen granules by immunohistochemical stains for trypsin. Acinar cell carcinoma may mimic pancreatic neuroendocrine tumors on cytomorphology and immunohistochemistry because they may occasionally stain with neuroendocrine markers [47]. In a small sample such as a cell block preparation, it is often difficult to prove its true lineage and may be erroneously diagnosed as a neuroendocrine tumor.

**Figure 16:**
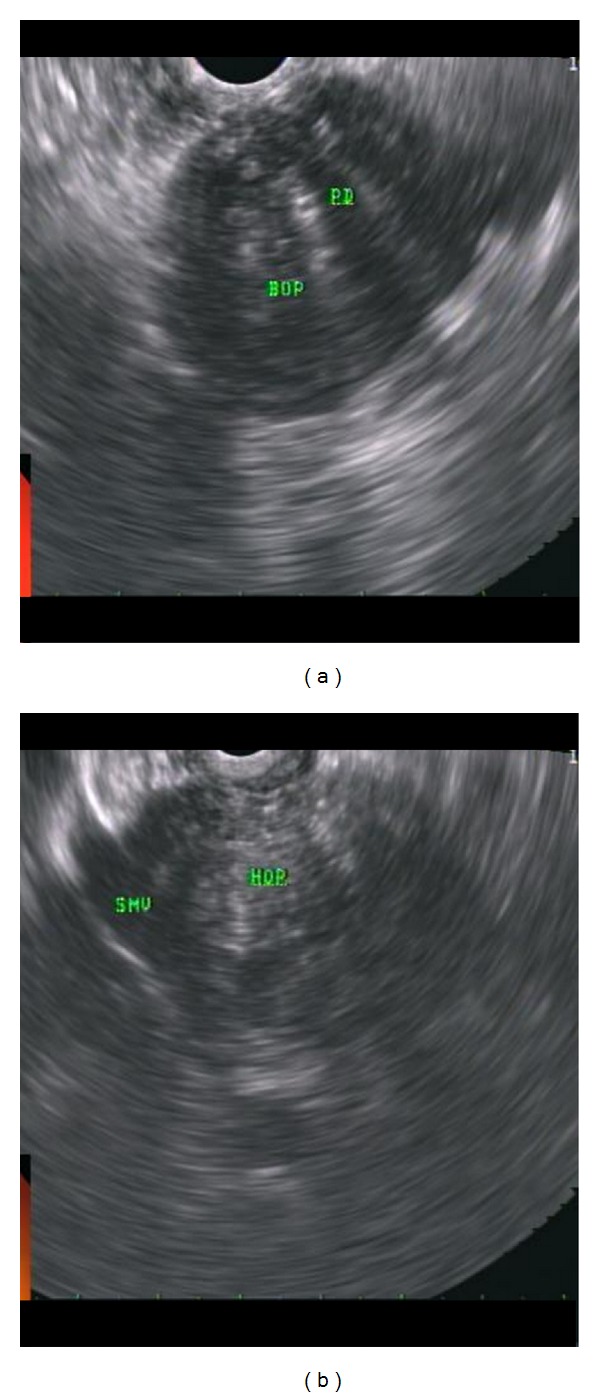
Autoimmune pancreatitis: EUS showed the entire pancreas to be diffusely enlarged, lobulated, and hypoechoic in appearance with coarse echogenic foci. The main PD was small in size. (a) and (b) showed the changes in body and head of pancreas, respectively. The proximal SMV (superior mesenteric vein) was encased and partially obstructed.

 (6) Autoimmune pancreatitis: a 44-year-old gentleman with new onset diabetes mellitus presented with obstructive jaundice and weight loss. ERCP showed common hepatic biliary stricture. Brushings were negative for malignancy. A plastic biliary stent was placed. CT abdomen showed mild intrahepatic biliary dilatation, stent in place, but no obvious mass lesion. EUS was then performed. The pancreas was diffusely enlarged lobulated, and hypoechoic in appearance with coarse echogenic foci. The main PD was small in size. The proximal superior mesenteric vein (SMV) was encased, and partially obstructed with portal collaterals (Figure 16). Four passes were done of the pancreatic neck with 19-gauge Quick core needle via transgastric approach. After confirmation of adequate cellularity, the specimen was submitted for cytologic and histologic analyses.

**Figure 17:**
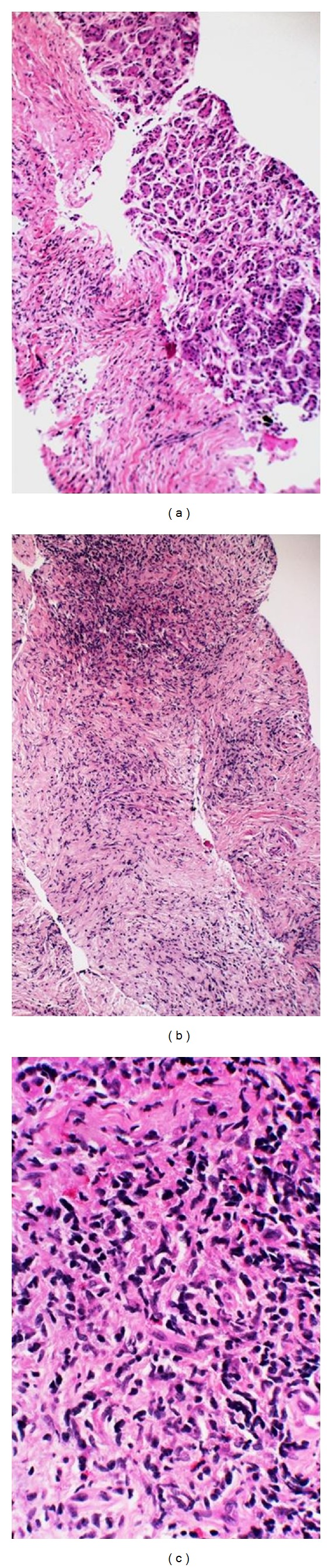
Autoimmune pancreatitis: sclerotic parenchyma compared to an intact acinar parenchyma, H&E cell block at 100x (a), infiltrated by inflammatory cells, H&E cell block at 100x (b), consisting predominantly of lymphocytes and rare eosinophils, H&E cell block at 100x (c).

Aspirate smears from sclerotic areas in autoimmune pancreatitis (AIP) may yield no cells; conversely, identifying ductal epithelium during onsite evaluation does not guarantee diagnostic tissue in the cell block. Therefore, there is only a limited role for onsite evaluation if this diagnosis is already radiologically and serologically (elevated serum IgG4) established. Typically, the role for cytopathologist during the EUS-FNA procedure in patients with AIP is to rule out a pancreatic malignancy in a mass lesion. For suspected AIP lesions, instead of an FNA, a core biopsy with cutting needle is preferable [48] for documenting sclerosis, pancreatic atrophy, and lymphoplasmacytic inflammation in the sclerotic areas, by means of a cell block (Figure 17). Inflammation is usually patchy and includes predominantly lymphocytes [49]. If sufficient numbers of plasma cells are present, IgG4 stain may be attempted on the cell block by immunohistochemistry.

**Figure 18:**
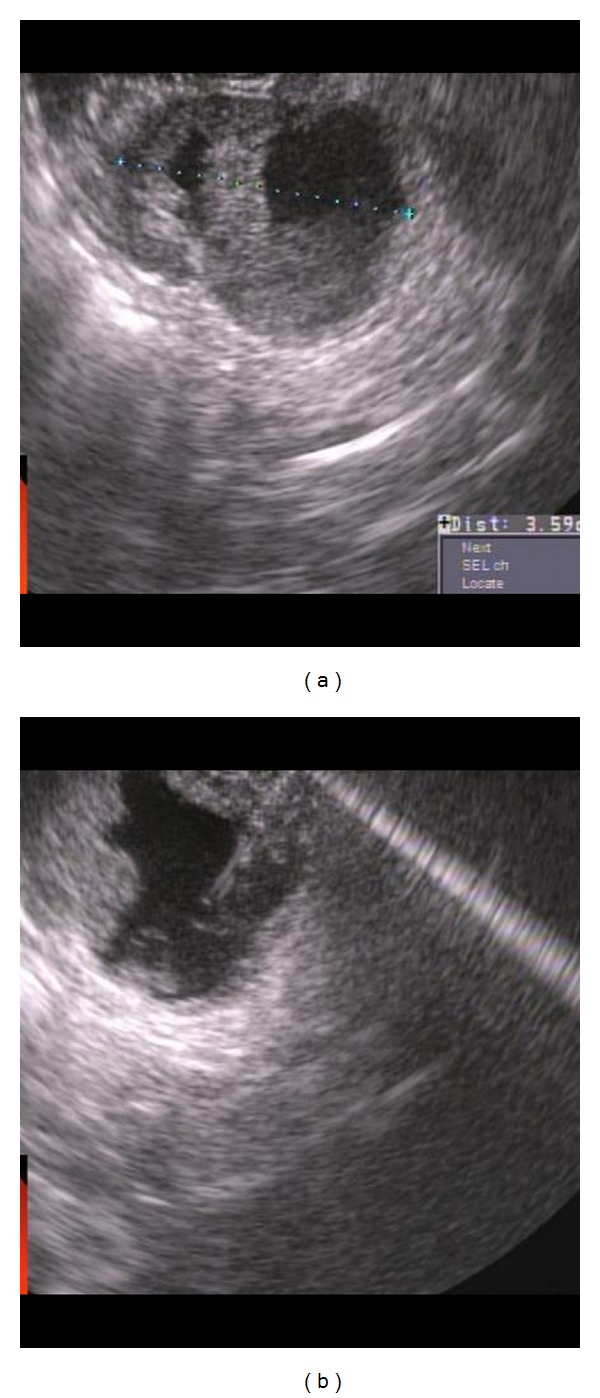
Solid pseudopapillary tumor. (a) EUS showed a 36 mm anechoic cystic lesion with a hypoechoic thick irregular rim and a solid polypoid component in the pancreatic head/body junction. (b) The cystic and solid components were separately FNA using a 19-gauge needle via transgastric approach.

 (7) Solid pseudopapillary tumor: a 43-year-old female with no significant past medical history presented with abdominal pain. CT abdomen showed a 3 cm cystic lesion in the head of pancreas. EUS showed a 36 mm anechoic irregular lesion with a hypoechoic thick irregular rim and a solid polypoid component in the pancreatic head/body junction (Figure 18). The cystic and solid components were separately aspirated using a 19-gauge needle via transgastric approach. The specimen adequacy was confirmed by an onsite cytopathologist.

**Figure 19:**
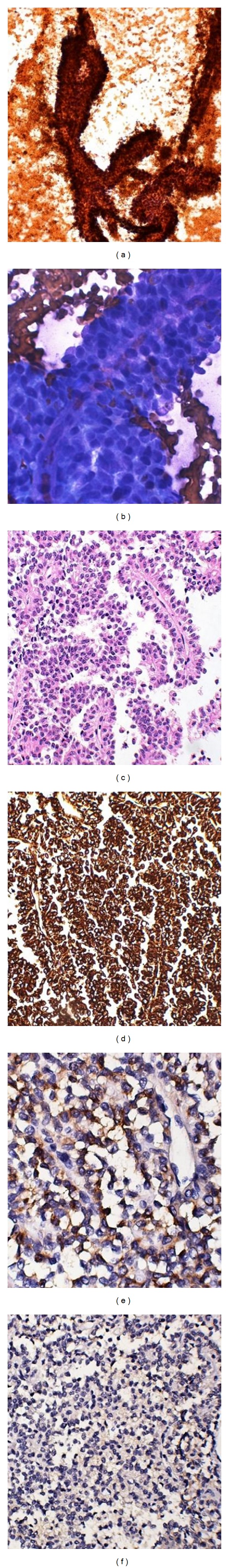
Solid pseudopapillary tumor: (a) slender pseudopapillary structures, Papanicolaou stain at 200x; (b) central fibrovascular cores, Diff-Quik stain at 400x; (c) cell block, H&E at 200x; immunoperoxidase stains (d) positive vimentin, 200x; (e) positive CD10, 400x; (f). negative cytokeratin Cam 5.2, 200x.

Onsite evaluation revealed markedly cellular aspirate smears with the cells arrayed along elongate papillary structures with central fibrovascular cores (Figure 19). Uniformly bland, dyscohesive cells were scattered between the papillae. There was no mucin or macrophages in the background. These two key features (papillae and bland dyscohesive cells) were the clues to a spot diagnosis of solid and cystic pseudopapillary tumor of pancreas [50]. Immunohistochemical profile of this tumor is interesting: mostly negative for cytokeratin, positive for vimentin and CD10 and nuclear staining for beta catenin [51].

**Figure 20:**
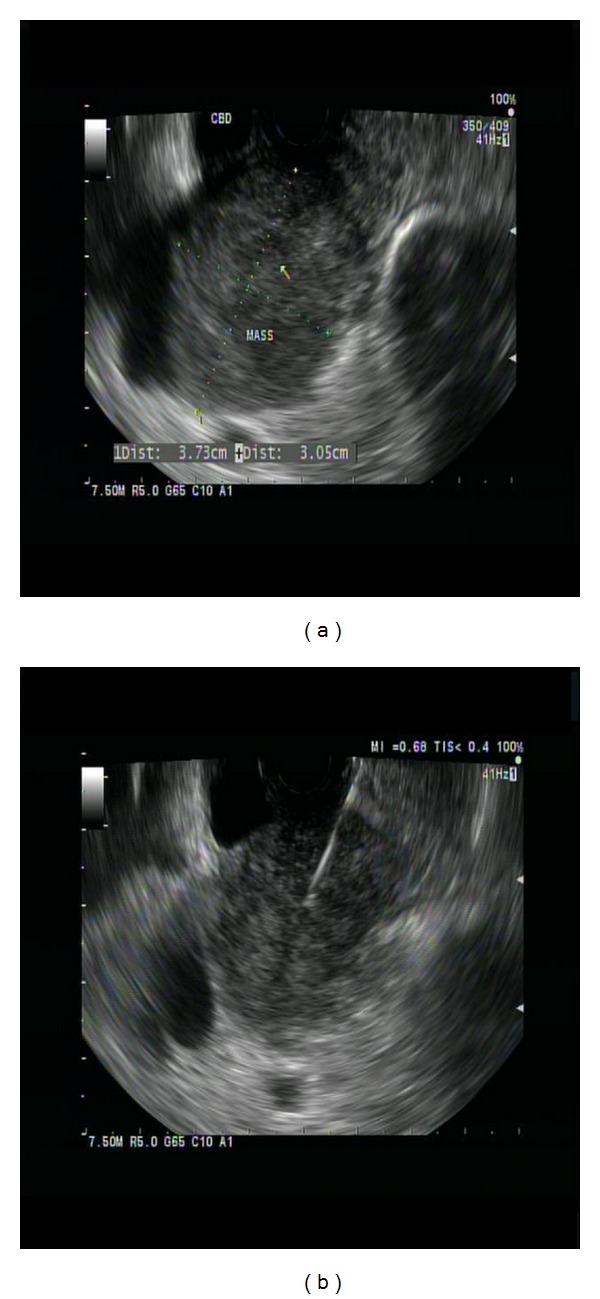
Metastatic lung cancer. (a) EUS showed a 37 mm by 30 mm well-defined, hypoechoic, and heterogeneous peripancreatic solid mass at the level of pancreatic head causing distal CBD stenosis. (b) Two FNA passes were done using 25-gauge needle via transduodenal approach.

 (8) Metastatic lung cancer An 80-year-old female with history of bilateral lung adenocarcinoma, surgical lobectomy, chemotherapy, and radiation in the past presented with new onset jaundice. CT abdomen showed a large 38 mm by 31 mm mass located in the peripancreatic area that was causing distal CBD stenosis. PET scan showed an increasing hypermetabolic focus in the same area, suggestive of neoplastic process. EUS showed a 37 mm by 30 mm well-defined, hypoechoic, and heterogeneous peripancreatic mass at the level of pancreatic head causing distal CBD stenosis (Figure 20). Two FNA passes were done using 25-gauge needle via transduodenal approach. The specimen was analyzed by onsite cytopathologist for adequate cellularity.

**Figure 21:**
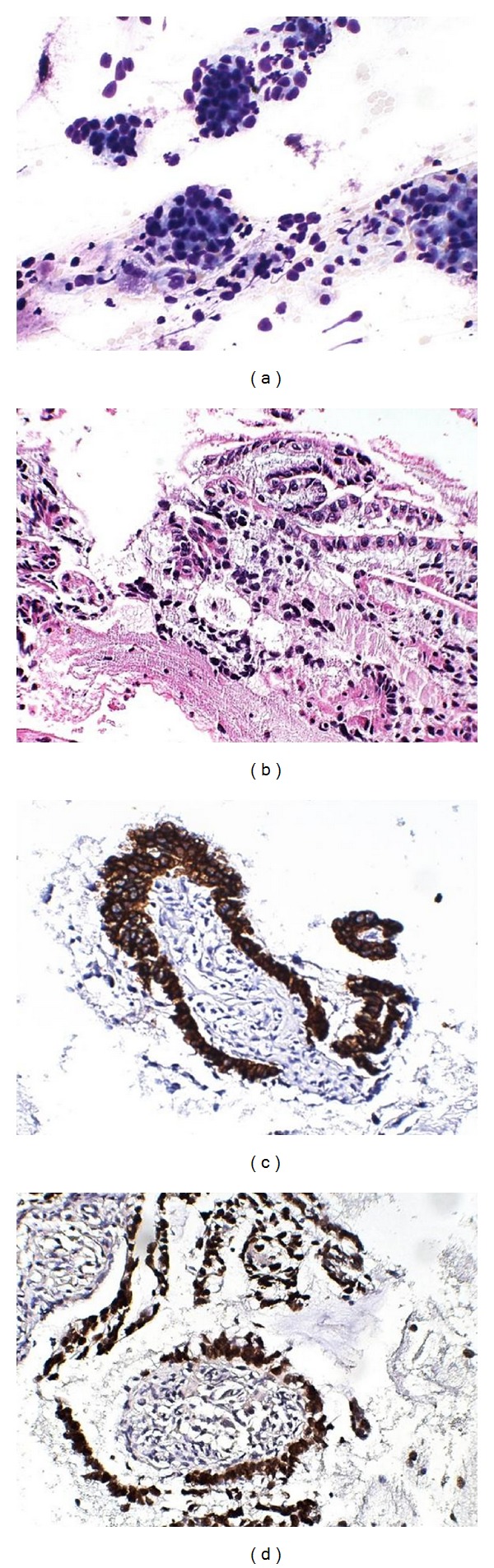
Lung metastasis to pancreas: (a) tumor cell clusters, Diff-Quik stain at 200x; (b) strips of tumor epithelium, H&E cell block at 200x; (c) cytoplasmic staining of cytokeratin CK7, immunoperoxidase stain, 400x; (d) nuclear staining of TTF1, immunoperoxidase stain, 200x.

The composite on Figure 21 shows adequate atypical clusters on immediate evaluation. It does not appear different from a pancreatic ductal adenocarcinoma. However, on immunohistochemistry, the tumor was CK7+/TTF1+/CK20−. This immunoprofile is consistent with the lung primary. A typical pancreatic primary would be CK7+/TTF1−/CK20+. Clinical history and cell block for immunohistochemistry are essential in arriving at the right diagnosis.

**Figure 22:**
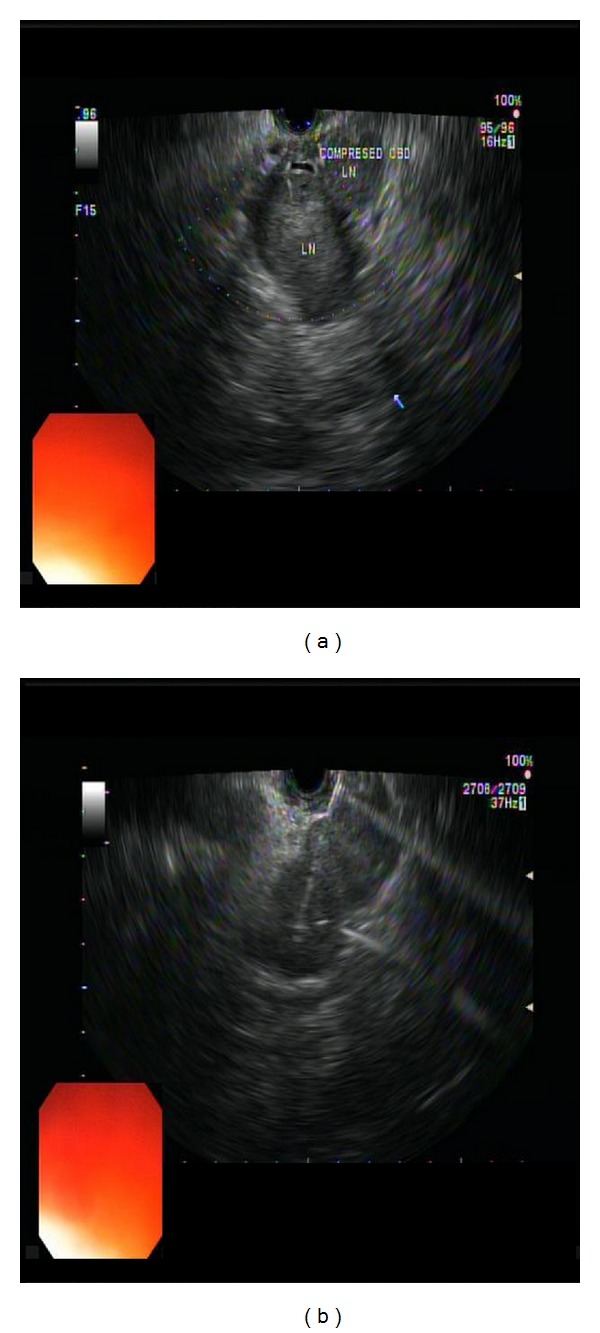
Renal metastasis to pancreas. (a) EUS showed multiple confluent, well-circumscribed hypoechoic, spherical masses measuring up to 40 mm in the peripancreatic (head) area compressing distal CBD. Multiple anechoic areas of necrosis were seen inside the lesions. (b) A single FNA pass was done using a 19-gauge needle via transduodenal approach.

 (9) Metastatic renal cell cancer: a 73-year-old woman was found to have multiple peripancreatic masses, left adrenal mass, and a left lower pole kidney mass on imaging studies. EUS showed multiple confluent, well-circumscribed hypoechoic, spherical masses, measuring up to 40 mm, in the peripancreatic (head) area compressing distal CBD. Multiple anechoic areas were noted within these mass, representing necroses (Figure 22). A single FNA pass was done using a 19-gauge needle via transduodenal approach. A 31 mm hypoechoic, well-circumscribed mass was noted in left adrenal. Its appearance was similar to other masses imaged. One FNA pass was done using 22-gauge needle via transgastric approach and submitted for cell block only. The peripancreatic mass was analyzed by onsite cytopathologist for adequate cellularity.

**Figure 23:**
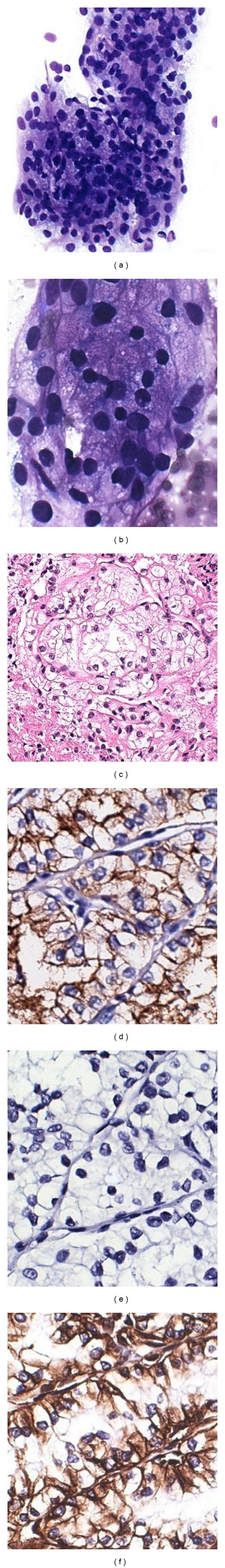
Renal metastasis to pancreas: (a) and (b) abundant, bubbly cytoplasm, Diff-Quik stain at 200x and 400x; (c) clear cytoplasm, outlined by sharp cell membrane, cell block H&E at 200x; (d) positive CD10 immunoperoxidase stain, at 400x; (e) negative cytokeratin CK7, at 400x; (f) positive vimentin immunoperoxidase stain, at 400x.

Onsite evaluation of the peripancreatic mass showed an extremely cellular aspirate on Diff Quick stain and was deemed adequate for evaluation. The clear cell morphology of the metastatic tumor is best appreciated on routine H&E stain of the cell block (Figure 23), least evident on air-dried smears for onsite evaluation, moderately so on alcohol fixed, Papanicolaou-stained smears. Cell blocks from both lesions (peripancreatic and left adrenal) showed identical morphology with clear cells. Immunoperoxidase stains are mandatory to differentiate the following: normal left adrenal cortical parenchyma/adrenal cortical adenoma versus a left clear cell renal carcinoma (RCC) in the upper pole. Normal adrenal cortical parenchyma cannot be distinguished from an adrenal cortical adenoma by immunoperoxidase stains but they can both be distinguished from an RCC. Synaptophysin, Melan 1, and inhibin are positive in adrenal cortical cells while CD10, vimentin, and RCC antigen highlight RCC and do not crossreact with adrenal cortex [52]. Based on the immunohistochemical profile, both masses were diagnosed as metastatic renal cell carcinoma to peripancreatic tissue and left adrenal.

## 7. Conclusion

In summary, endoscopic ultrasound is the most sensitive imaging modality for solid pancreatic lesions. It is an outpatient safe procedure, with high accuracy of 78–94% for T (local tumor) stage and 64–82% for N (lymph node) stage. The specificity is low (about 75%), but can be increased to 100% with an accuracy of 95% with the addition of fine needle aspiration. The diagnostic yield of EUS-FNA for solid pancreatic lesion is 86.8–98.5% for cytologic and 68.9–89% for histologic examinations, while 65–100% for lymph nodes. Factors related to either endoscopist or cytopathologist affect the diagnostic yield of EUS-FNA. A close interaction with cytopathologist is vital in improving the diagnostic yield. The final diagnosis is based upon correlation of clinical, EUS, and cytologic features.
